# Effects of multiple low-dose intranasal dexmedetomidine administration on postoperative pulmonary complications in elderly patients undergoing thoracoscopic radical resection of lung cancer: a single-center randomized controlled trial

**DOI:** 10.3389/fphar.2026.1914046

**Published:** 2026-10-02

**Authors:** Bing Li, Yao Liu, Xinmin Liu, Mengyan Han, Xing Meng, Mingyang Sun, Jiaqiang Zhang, Wei Zhang

**Affiliations:** 1 Department of Anesthesiology and Perioperative Medicine, Henan Provincial People’s Hospital, Zhengzhou, China; 2 Department of Anesthesiology, People’s Hospital of Zhengzhou, Zhengzhou, China

**Keywords:** dexmedetomidine, elderly patients, lung cancer, multiple low doses, nasal spray, postoperative pulmonary complications

## Abstract

**Background:**

Postoperative pulmonary complications (PPCs) ranging from 30% to 50% have been recognized as a major threat to postoperative recovery. Current conventional interventions cannot effectively reduce the occurrence of PPCs.

**Objective:**

In this study, we aimed to investigate the effects of multiple low-dose intranasal dexmedetomidine administration on PPCs in elderly patients.

**Methods:**

In this double-blind, randomized, controlled trial, elderly patients were randomly assigned at a 1:1 ratio to receive multiple low-dose intranasal dexmedetomidine administration (MD group) or normal saline nasal spray (NS group). The primary outcome was the incidence of PPCs within 7 days after surgery. Key secondary outcomes included the severity grading and classification of PPCs. Other secondary outcomes included serum interleukin-6 (IL-6) and tumor necrosis factor-α (TNF-α) levels, Richards-Campbell sleep questionnaire (RCSQ) scores at predefined time points, and adverse reactions.

**Results:**

A total of 190 patients were screened, and 186 completed the study. The incidence of PPCs was significantly lower in the MD group than in the NS group (9.68% vs. 22.58%, *P* = 0.017), with a risk ratio (RR) of 2.333 [95% confidence interval (CI): 1.173–6.317], absolute risk reduction (ARR) of 12.90%, and number needed to treat (NNT) of 7.75. Sensitivity analyses using the per-protocol population (PP) dataset yielded consistent results, confirming the stability and reliability of the primary outcome. The distribution of severity grading of PPCs also differed significantly between the groups (*P* = 0.036). After Bonferroni correction, the between-group difference in PPCs severity grading was not statistically significant (corrected *P* = 0.072). The distribution of pneumonia also differed significantly between the groups (*P* = 0.029). There were significant group effects, time effects, and interaction effects for IL-6, TNF-α, and RCSQ in the two groups after surgery (all *P* < 0.05). No significant between-group differences were observed in drug-related adverse events.

**Conclusion:**

Multiple low-dose intranasal dexmedetomidine administration significantly reduces the incidence of PPCs in elderly patients undergoing radical thoracoscopic lung-cancer resection with a favorable safety profile.

**Clinical Trial Registration:**

https://www.chictr.org.cn/showproj.html?proj=243165; identifier ChiCTR2400090593.

## 1 Introduction

Lung cancer remains the most commonly diagnosed malignancy and the leading cause of cancer-related mortality in China (Hemsinli et al., 2022). Surgical resection, particularly video-assisted thoracoscopic surgery, is the cornerstone of curative treatment for early-stage lung cancer. However, thoracic surgery is associated with a high incidence of postoperative pulmonary complications (PPCs), ranging from 30% to 50% in elderly patients (Fernandez-Bustamante et al., 2017a). PPCs are major contributors to prolonged hospital stay, increased medical costs, elevated perioperative morbidity, and even early mortality. Even mild respiratory events, such as transient hypoxemia or prolonged oxygen supplementation, can substantially impair postoperative recovery. Therefore, effective and safe strategies to reduce PPCs are urgently needed in perioperative care, especially for elderly patients undergoing thoracoscopic lung-cancer resection.

Dexmedetomidine is a highly selective *α*_2_-adrenergic agonist with well-established sedative, anxiolytic, analgesic-sparing, and anti-inflammatory properties. Unlike conventional sedatives, dexmedetomidine provides cooperative sedation without significant respiratory depression, making it particularly suitable for elderly populations. Accumulating evidence indicates that intraoperative intravenous dexmedetomidine exerts potent bronchodilatory activity by inhibiting cholinergic nerve-mediated tracheal smooth muscle contraction. This airway-relaxing effect alleviates intraoperative airway spasm, reduces peak inspiratory pressure, and improves oxygenation during one-lung ventilation (Mikami et al., 2017; Bai et al., 2021; Yang et al., 2023); attenuates surgical stress-induced inflammatory responses; alleviates ventilator-associated lung injury (Zhu et al., 2020); improves respiratory mechanics (Xu et al., 2020; Wang et al., 2025); and reduces the risk of PPCs in patients undergoing thoracic surgery (Xie et al., 2020; Xu et al., 2023; Zorrilla-Vaca et al., 2024). The underlying pulmonary protective mechanisms may involve inhibition of oxidative stress, suppression of proinflammatory cytokine release, attenuation of sympathetic overactivation, and inhibition of neutrophil infiltration and aggregation (Xie et al., 2020). Nevertheless, the predominant route of administration in clinical practice and most studies is intravenous infusion.

Oral dexmedetomidine undergoes extensive hepatic first-pass elimination, leading to very low systemic bioavailability and unstable efficacy (Li et al., 2024). Although patients undergoing thoracoscopic radical lung-cancer resection routinely require intraoperative venous access, perioperative intranasal dexmedetomidine administration provides favorable bioavailability and predictable pharmacokinetic profiles versus oral and intravenous administration (Kuang et al., 2022; Anttila et al., 2003). Its main strengths are reduced hemodynamic fluctuations (Bhalotra et al., 2024) and milder respiratory depression (Li et al., 2024). Moreover, this non-invasive, convenient, patient-friendly route is independent of venous access and lowers medical resource consumption (Bhargavi et al., 2023). Previous studies have demonstrated that intranasal dexmedetomidine administration leads to rapid absorption, satisfactory sedation, and a good safety profile (Kuang et al., 2022; Wang et al., 2024; Wang et al., 2025). Furthermore, intranasal delivery avoids the hypotension and bradycardia commonly associated with intravenous bolus or infusion, thereby improving hemodynamic stability (Bhalotra et al., 2024). However, nearly all available clinical studies (Wang et al., 2023; Li et al., 2024; Gui et al., 2026) are limited to single-dose regimens. A single intranasal dose may provide insufficient duration of effect to cover the high-risk period for PPCs, which typically peaks within 24 h–48 h postoperatively. In addition, single high doses may exceed the nasal mucosal absorption capacity and increase the risk of over-sedation. Accordingly, a multiple low-dose intranasal regimen may offer sustained drug exposure and better protection against PPCs; yet, supporting clinical evidence remains scarce.

Based on the above evidence, we hypothesized that multiple low-dose intranasal dexmedetomidine administration initiated preoperatively and continued postoperatively could provide stable and sustained sedation, suppress excessive inflammatory responses, improve sleep quality, and ultimately reduce the incidence of PPCs in elderly patients undergoing radical thoracoscopic lung-cancer resection. The present randomized, double-blind, controlled trial was designed to evaluate the efficacy and safety of this non-invasive regimen for preventing PPCs.

## 2 Materials and methods

### 2.1 Study subjects

Elderly patients undergoing elective radical thoracoscopic lung-cancer resection at Henan Provincial People’s Hospital between 1 January 2025 and 31 October 2025 were enrolled in this study. Written informed consent was obtained from all participants prior to enrollment. The study protocol was approved by the Ethics Committee of Henan Provincial People’s Hospital on 23 September 2024 [(2024) Ethics Review No. 165] and registered with the Chinese Clinical Trial Registry (https://www.chictr.org.cn; Registration No. ChiCTR2400090593) on 30 December 2024. The study adhered to the Consolidated Standards of Reporting Trials guidelines and principles of the Declaration of Helsinki. All enrolled patients provided written informed consent before surgery.

Inclusion criteria: age ≥65 years; undergoing elective radical thoracoscopic lung-cancer resection; either gender; body mass index (BMI) 18 kg/m^2^–24 kg/m^2^; American Society of Anesthesiologists (ASA) physical status II–III; scheduled as the first case of the day; estimated surgery duration >1 h. Exclusion criteria: history of prior thoracic surgery or perioperative radiotherapy; complicated with obstructive sleep apnea hypopnea syndrome, depression, and insomnia, with sedative medication use within recent 3 months, respiratory cysts, tumors or polyps, acute/chronic rhinitis, persistent nasal congestion, nasal polyps, deviated nasal septum and recent nasal surgery/trauma, damaged nasal mucosa and obstructed nasal cavities, or upper respiratory tract infection within 2 weeks prior to enrollment; severe bradycardia, sick sinus syndrome, second-degree or higher atrioventricular block without permanent pacemaker implantation, ongoing beta-blocker administration, or severe heart failure with left ventricular ejection fraction less than 30%; preoperative forced expiratory volume in one second below 80% of the predicted value or history of severe asthma; severe hepatic or renal dysfunction; intraoperative surgical conversion, unplanned reoperation in the post-anesthesia care unit (PACU), or postoperative intensive care unit admission; hypersensitivity to study drugs; administration of analgesics, antiemetics, or glucocorticoids within 24 h before surgery; participation in other clinical trials within 3 months before enrollment; severe cognitive, verbal, auditory, or visual dysfunction, mental disorders, coma, and other conditions interfering with study compliance.

### 2.2 Procedure

Based on a computer-generated randomized number, elderly patients were randomly allocated in a 1:1 ratio to the multiple low-dose intranasal dexmedetomidine administration group (group MD, Jiangsu Hengrui Medicine Co., Ltd.) and the normal saline group (group NS, the NS group received normal saline in identical spray devices). Surgeons, anesthesiologists, outcome assessors, and patients were blinded to group allocation. Data analysis was performed by a statistician who was not involved in patient enrollment or intervention. The most common drug-related adverse events of intranasal dexmedetomidine administration include hypotension, bradypnea, hypoxemia, excessive sedation, and bradycardia (Kuang et al., 2022; Wang et al., 2025). In the event of an emergency, the on-call physician was authorized to unblind the group allocation to initiate appropriate treatment as needed.

Interventions were initiated on the evening before the surgery according to the patient’s number. Specially assigned personnel prepared the two study drugs in identical-appearing spray devices, which were then sealed in sequentially numbered envelopes. All patients received nasal spray administered at 20:00 nightly from the night before the surgery until postoperative day 3. Patients in group MD received a total of 75 μg of intranasal dexmedetomidine administration daily. Patients in group NS received an equal volume of normal saline nasal spray at the same time points by the same procedure. The standardized administration procedure of nasal spray was as follows: when multiple sprays were required in the same nostril, patients were instructed to tilt their head backward at 20°–30° after each spray and inhale for 30 s before the next application, repeating until the full dose was completed. All procedures were completed within 3 min. The drug-related adverse events were monitored for 60 min after each intervention, including hypotension (systolic blood pressure <90 mmHg or diastolic blood pressure <50 mmHg for 5 min or longer), excessive sedation (Ramsay score ≥5), bradycardia (heart rate <50 bmp), and respiratory depression (respiratory rate <8 bmp or SpO_2_ < 92%). Adverse events were managed as part of routine care and documented by a dedicated post-administration adverse event assessor. Emergency un-blinding documents were filed in a sealed form and could only be accessed if severe adverse reactions occurred and treatment adjustment was mandatory. All dedicated drug administrators, dedicated post-administration adverse-event assessors, dedicated post-administration outcome assessors, and clinical medical staff were kept blinded to the treatment allocation.

Anesthesia induction and maintenance: Preoperatively, peripheral venous access was established, and vital signs (HR, SpO_2_, NIBP, and ECG) were monitored. Ultrasound-guided internal jugular vein and radial artery cannulation were performed. Upon admission to the operating room, 5 mg dexamethasone and 0.5 mg penehyclidine hydrochloride were administered intravenously. Anesthesia induction was performed as follows: intravenous administration of midazolam 0.03 mg/kg–0.05 mg/kg, sufentanil 0.3 μg/kg–0.5 μg/kg, etomidate 0.1 mg/kg–0.4 mg/kg, and rocuronium 0.6 mg/kg–1.0 mg/kg. Following successful double-lumen endotracheal intubation (35 Fr tube for female patients and 37 Fr tube for male patients), anesthesia was maintained with intravenous infusion of propofol combined with remifentanil to keep the bispectral index (BIS) between 40 and 60. Intraoperative cisatracurium was supplemented as needed to maintain adequate muscle relaxation. Twenty minutes before the end of surgery, 5 mg tropisetron was administered intravenously.

Intraoperative management: During one-lung ventilation, all patients received pressure-controlled ventilation-volume guaranteed (PCV-VG). The target tidal volume was set to 4 mL/kg–6 mL/kg ideal body weight with positive end-expiratory pressure (PEEP) maintained at 5 cm H_2_O, and peak airway pressure (*P*_peak_) was strictly limited to a maximum of 30 cm H_2_O; if *P*_peak_ was exceeded, volume-controlled ventilation (VCV) was switched to pressure-controlled ventilation (PCV) (Byun et al., 2018; Li et al., 2020; Yao et al., 2020). Recruitment maneuvers were performed, and the procedural protocol involved stepwise incremental PEEP from 5 to 8, 10, and 12 cm H_2_O, with each pressure level maintained for 30 s–40 s. The plateau airway pressure was strictly maintained below 30 cm H_2_O throughout the maneuver. After adequate lung recruitment, PEEP was gradually reduced back to 5 cm H_2_O, and conventional lung-protective ventilation parameters were resumed.

Hemodynamic goals: MAP/HR fluctuations ≤20% of baseline; 2 μg/min–12 μg/min norepinephrine was administered when MAP decreased by more than 20%; a 0.2-mg bolus of nicardipine was given when MAP increased by more than 20%; 0.3 mg atropine was used when HR decreased by more than 20%.

Postoperative analgesia: 0.5 mg/kg oxycodone and 10 mg tropisetron were diluted to 100 mL (background infusion: 0 mL/h, bolus: 3 mL, and lockout: 20-min).

Anesthesia recovery: consistent with the routine protocol of our center, tracheal extubation for all enrolled thoracic surgical patients was performed in the PACU. Neuromuscular blockade antagonists were administered in the PACU, and standardized extubation assessment (including spontaneous ventilation, consciousness recovery, and airway protective reflex evaluation) was completed here. Patients with a Steward’s awakening score ≥4 and stable vital signs were transferred to the ward. Rescue medications were given for pain/postoperative nausea and vomiting (PONV) by ward physicians.

### 2.3 Demographics and perioperative variables

Baseline data included age, sex, BMI, ASA, history of hypertension, history of diabetes mellitus, smoking history, history of motion sickness or PONV, history of non-thoracic surgery, preoperative anxiety score, history of insomnia, total fluid infusion volume, intraoperative norepinephrine dosage, intraoperative atropine dosage, intraoperative sufentanil consumption, intraoperative remifentanil consumption, intraoperative propofol consumption, intraoperative blood loss, urine output, operative duration, anesthesia duration, and length of stay in the PACU.

### 2.4 Outcomes

The primary outcome of this study was the overall incidence of PPCs within 7 days after surgery (PPCs were defined according to Supplementary Table S1) (Fernandez-Bustamante et al., 2017a). Key secondary outcome 1 is the grading of PPCs [according to the international standard for PPCs, the EPCO classification (European Perioperative Clinical Outcome) adopts a 5-grade scoring system ranging from grade 0 to grade 4: grade 0: no postoperative pulmonary complications; grade 1: mild PPCs (only basic symptomatic treatment required); grade 2: moderate PPCs (oxygen therapy needed without mechanical ventilation); grade 3: severe PPCs (non-invasive ventilation or thoracic catheter drainage required); grade 4: critical PPCs (invasive mechanical ventilation or death related to pulmonary complications)](Jammer et al., 2015; Fernandez-Bustamante et al., 2017b; Abbott et al., 2018). Key secondary outcome 2 is the classification of PPCs. Other outcomes included the levels of interleukin-6 (IL-6) and tumor necrosis factor-*α* (TNF-α) at preoperative (T1), 24 h (T2), 48 h (T3), and 72 h (T4) after surgery; intraoperative use of vasoactive drugs; use of rescue analgesics and antiemetics; sleep quality scores assessed by the Richards–Campbell sleep questionnaire (RCSQ) at T1, T2, T3, and T4; PONV at 72 h after surgery; bradycardia, hypotension, excessive sedation, and hypoxemia.

## 3 Sample size

In this prospective, double-blind, randomized controlled trial, elderly patients were randomly allocated in a 1:1 ratio to the multiple low-dose intranasal dexmedetomidine administration group (group MD) and the normal saline group (group NS) according to a computer-generated randomized number. The primary endpoint was defined as the incidence of PPCs within 7 days after surgery. Sample size calculation was performed based on our preliminary trial data and previous published literatures (Xie et al., 2020). Sample size was calculated based on an assumed PPCs incidence of 12% in the MD group and 30% in the NS group, and a total of 162 participants were required for sample size calculation under a two-sided test with a significance level of *α* = 0.05 and statistical power of 80%. After accounting for an anticipated 15% dropout rate, the final sample size was determined to be 190 participants in both groups combined.

## 4 Statistical analysis

Statistical analyses were performed using SPSS 27.0 software. The Shapiro–Wilk test was used to assess the normality of continuous variables. Normally distributed data were presented as the mean ± standard deviation (Mean ± SD). Repeatedly measured data were analyzed with generalized estimating equations (GEE). An exchangeable or AR (1) correlation structure was adopted. The main effects of group and time, along with their interaction effect, were evaluated. A two-tailed *P* < 0.05 was considered statistically significant. When significant interactions were detected, *post hoc* pairwise comparisons at each time point were performed using independent-samples *t*-test with Bonferroni correction to control for type-I error. Non-normally distributed data were reported as median (interquartile range, median (IQR)), with between-group comparisons using the Mann–Whitney *U*-test. Categorical variables were described as frequencies (percentages). Between-group comparisons of nominal categorical variables were performed using the Pearson *χ* ^2^-test or Fisher’s exact test, as appropriate. Ordinal categorical variables were analyzed using the Mann–Whitney *U*-test. A two-sided *P* < 0.05 was considered statistically significant. A fixed-sequence procedure was prespecified in the study protocol and statistical analysis plan (SAP) before unblinding to control the family-wise error rate (FWER) and ensure the validity of confirmatory inference. No *post hoc* modifications to the testing sequence or statistical methods were permitted. All confirmatory endpoints shared the same two-sided significance level of *α* = 0.05 without alpha splitting. Testing proceeded in the prespecified hierarchical order: a statistically significant result (*P* ≤ 0.05) for the preceding endpoint was required before the next key secondary endpoint could be formally tested. All randomized patients were included in the intention-to-treat (ITT) analysis regardless of protocol deviations, in accordance with the ITT principle. Sensitivity analysis was performed using the per-protocol (PP) population or modified intention-to-treat (mITT).

## 5 Results

### 5.1 Baseline and perioperative characteristics

A total of 190 patients were enrolled in this trial. Four patients withdrew informed consent before receiving study intervention and withdrew from the trial. All randomized participants were included in the ITT analysis. A total of 186 patients (93 per group) finished the trial (Figure 1). No significant between-group differences were observed in the baseline characteristics or perioperative variables (all *P* > 0.05, Table 1).

**FIGURE 1:**
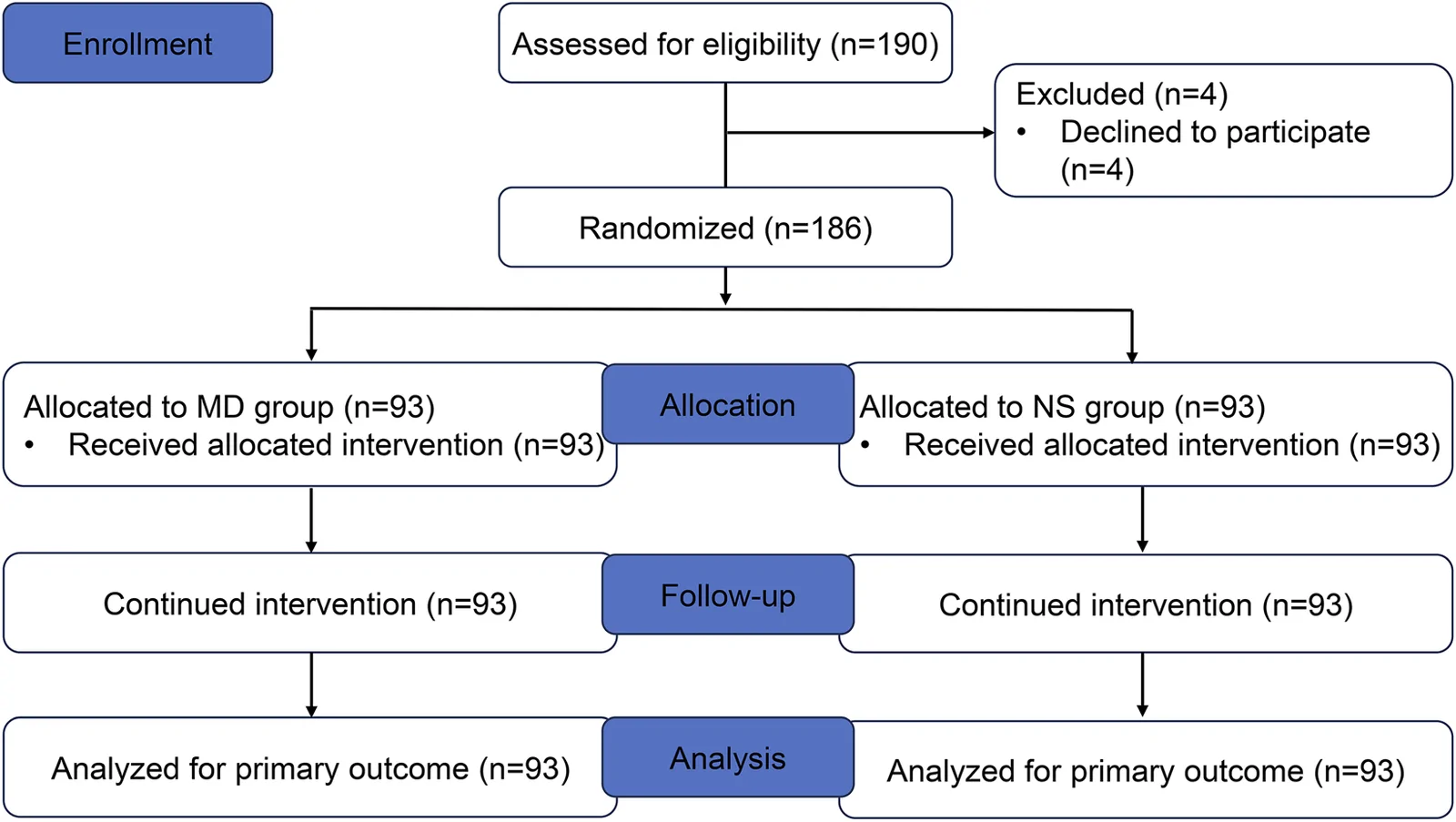
Flow diagram of the progress through the phases of a randomized trial of two groups.

**TABLE 1:** Patients characteristics and intraoperative baseline data.

Index	NS group (n = 93)	MD group (n = 93)	Value *t/χ* ^*2*^ */Z*	*P*
Age (year)	67 ± 8	69 ± 8	−1.705	0.089
BMI (kg/m^2^)	21 ± 2	21 ± 1	0.000	1.000
Gender	​	​	0.090	0.766
Male	55 (59.14%)	53 (56.99%)	​	​
Female	38 (40.86%)	40 (43.01%)	​	​
ASA	​	​	0.120	0.726
II	71 (76.34%)	73 (78.49%)	​	​
III	22 (23.66%)	20 (21.51%)	​	​
Hypertension history (yes)	18 (19.35%)	24 (25.81%)	1.110	0.293
Diabetes history (yes)	10 (10.75%)	8 (8.60%)	0.250	0.620
Smoking history (yes)	40 (43.01%)	38 (40.86%)	0.090	0.767
History of motion sickness or PONV (yes)	14 (15.05%)	16 (17.20%)	0.160	0.690
History of nonthoracic surgery (yes)	24 (25.81%)	26 (38.71%)	0.110	0.741
History of insomnia (yes)	34 (36.56%)	32 (34.41%)	0.090	0.759
Preoperative anxiety score	16 ± 4	15 ± 5	1.510	0.133
Intraoperative crystalloids (mL)	802 ± 57	810 ± 64	−0.900	0.369
Intraoperative blood loss (mL)	58 ± 11	55 ± 14	1.610	0.109
Urine output (mL)	311 ± 21	309 ± 29	0.540	0.590
Surgical duration(min)	138 ± 39	135 ± 34	0.540	0.590
Anesthesia time (min)	150 ± 38	146 ± 33	0.540	0.588
Sufentanil consumption (μg)	36 ± 8	35 ± 6	0.680	0.496
Remifentanil consumption (μg)	735 ± 35	740 ± 31	−0.730	0.467
Propofol dosage (mg)	388 ± 20	383 ± 18	1.790	0.074
Norepinephrine (μg)	560 (450, 600)	555 (450, 628)	0.320	0.753
Atropine (mg)	0.00 (0.00, 0.00)	0.00 (0.00, 0.00)	0.000	1.000
PACU stay duration (min)	109 ± 32	111 ± 24	−0.480	0.635

Data are presented as the mean (SD), n (%), or median. SD, standard deviation; BMI, body mass index; ASA, American Society of Anesthesiologists; PONV, postoperative nausea and vomiting; PACU, postoperative care unit.

### 5.2 Primary outcome

The overall incidence of PPCs within 7 days after surgery was significantly lower in the MD group (9.68%) than in the NS group (22.58%) (*P* = 0.017, Table 2). The risk ratio (RR) was 2.333 (95% CI: 1.173–6.317), the absolute risk reduction (ARR) was 12.90%, and the number needed to treat (NNT) was 7.75. Analyses based on the ITT and PP datasets produced consistent results for the overall incidence of PPCs, relative risk, absolute risk reduction, and NTT. The RR was 2.333 (95% CI: 1.173–6.317), the ARR was 12.90%, and the NNT was 7.75 (Table 3). Sensitivity analyses using the PP dataset yielded consistent results, confirming the stability and reliability of the primary outcome. Sensitivity analyses were only performed for the primary outcome. Given complete follow-up and no missing data, additional sensitivity tests were not conducted for secondary outcomes.

**TABLE 2:** Incidence, grading, and classification of PPCs within 7 days after surgery.

Index	NS group (n = 93)	MD group (n = 93)	Value *χ* ^*2*^	*P*
PPCs (%)	22.58	9.68	5.723	0.017*
Grading	​	​	6.625	0.036*
Grade 0 (%)	16.13	8.60	​	​
Grade 1 (%)	6.45	1.08	​	​
Grade 2 (%)	0	0	​	​
Grade 3 (%)	0	0	​	​
Grade 4 (%)	0	0	​	​
Bonferroni	​	​	6.625	0.072
Classification				
Pneumonia (%)	18.28	7.53	4.784	0.029*
Atelectasis (%)	4.30	2.15	0.689	0.407
Respiratory failure (%)	0	0	NA	NA
Pulmonary embolism (%)	0	0	NA	NA
Bronchopleural fistula (%)	0	0	NA	NA
Pleural empyema (%)	0	0	NA	NA

Data are presented as n (%), *P < 0.05. PPCs, postoperative pulmonary complications; NA, not applicable.

**TABLE 3:** Incidence of PPCs and the efficacy indices of the primary outcome between the two groups.

Index	*P*	RR	95% CI	ARR (%)	NNT
PPCs (%) (ITT)	0.017*	2.333	1.173–6.317	12.90	7.75
PPCs (%) (PP)	0.017*	2.333	1.173–6.317	12.90	7.75

Data are presented as n (%), *P < 0.05. PPCs, postoperative pulmonary complications; ITT, intention-to-treat; PP, per-protocol population; RR, risk ratio; CI, confidence interval; ARR, absolute risk reduction; NNT, number needed to treat.

### 5.3 Key secondary outcome

#### 5.3.1 Key secondary outcome 1: severity grading of PPCs

The distribution of PPC severity grades differed significantly between groups (*P* = 0.036, Table 2). After Bonferroni correction for multiple testing as prespecified, the corrected *P*-value was 0.072 (Table 2).

#### 5.3.2 Key secondary outcome 2: the classification of PPCs

The distribution of pneumonia differed significantly between the groups (*P* = 0.029, Table 2). In accordance with the fixed-sequence testing rule, formal inferential statistics for this endpoint were not performed owing to the non-significant corrected result for PPC severity.

### 5.4 Other outcomes

#### 5.4.1 Serum scores of IL-6, TNF-α, and the RCSQ at different time points

Repeated perioperative IL-6, TNF-α, and RCSQ data were analyzed via GEE. Significant group, time, and interaction effects were found for all indicators (all *P* < 0.05; Supplementary Table S2 and Table 4). No intergroup differences existed at baseline T1 (*P* > 0.05). In both groups, inflammatory factors rose rapidly and then declined (Figures 2A,B), while RCSQ scores dropped initially and then recovered gradually (Figure 2C). From T2 to T4, the MD group had lower inflammatory levels and higher RCSQ scores relative to the NS group.

**TABLE 4:** GEE analysis results of IL-6, TNF-α, and RCSQ.

Index	Effect	*β* coefficient	95% CI	*P*
IL-6	Group effect	−0.826	−1.347, −0.305	0.002
	Time effect	1.241	0.683, 1.799	<0.001
	Group × time interaction	−0.473	−0.891, −0.055	0.023
TNF-α	Group effect	−0.619	−1.072, −0.166	0.007
	Time effect	0.954	0.418, 1.490	<0.001
	Group × time interaction	−0.368	−0.724, −0.012	<0.043
RCSQ	Group effect	1.537	0.842, 2.232	<0.001
	Time effect	−1.125	−1.764, −0.486	<0.001
	Group × time interaction	0.682	0.135, 1.229	<0.015

GEE was used for statistical analysis; GEE, generalized estimating equations; IL-6, interleukin-6; TNF-α, tumor necrosis factor-α; RCSQ, Richards–Campbell sleep questionnaire.

**FIGURE 2:**
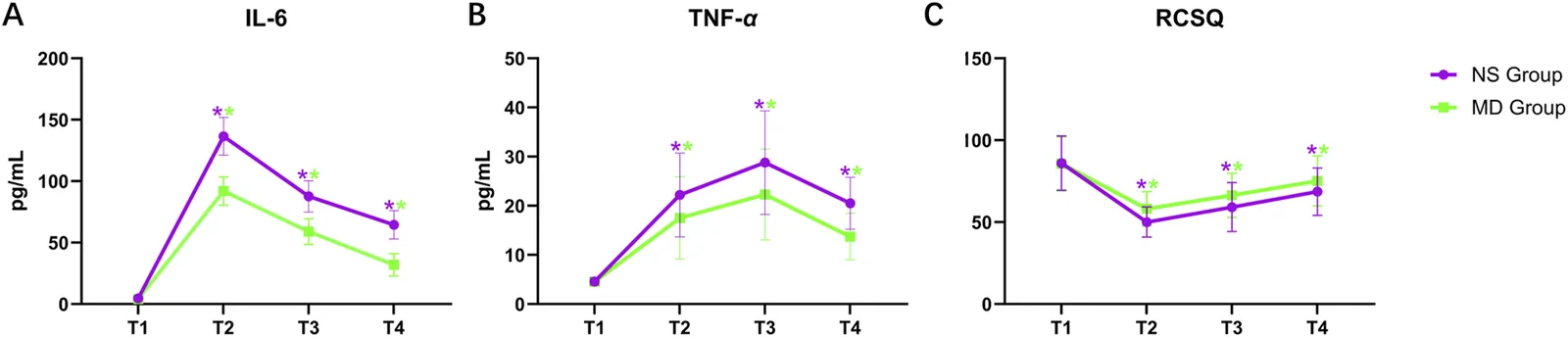
Dynamic variation of IL-6, TNF-α, and RCSQ scores in two groups. From left to right, each represents a different indicator: **(A)** for IL-6, **(B)** for TNF-α, and **(C)** for RCSQ. Data are expressed as the mean ± standard deviation (SD). Statistical analysis was performed by GEE. The main effects of group and time, along with their interaction effect, were evaluated. *P < 0.05. IL-6, interleukin-6; TNF-α, tumor necrosis factor-α; RCSQ, Richards-Campbell sleep questionnaire; T1, preoperative; T2, 24 h postoperatively; T3, 48 h postoperatively; T4, 72 h postoperatively; GEE, generalized estimating equations.

Intergroup comparisons were performed using the independent samples *t*-test after statistically significant differences were observed between the two groups via GEE. The IL-6 and TNF-α levels at T2, T3, and T4 in the MD group were significantly lower than that in the NS group (Figures 3A,B). The RCSQ scores in the MD group at T2, T3, and T4 were significantly higher than those in the NS group (Figure 3C).

**FIGURE 3:**
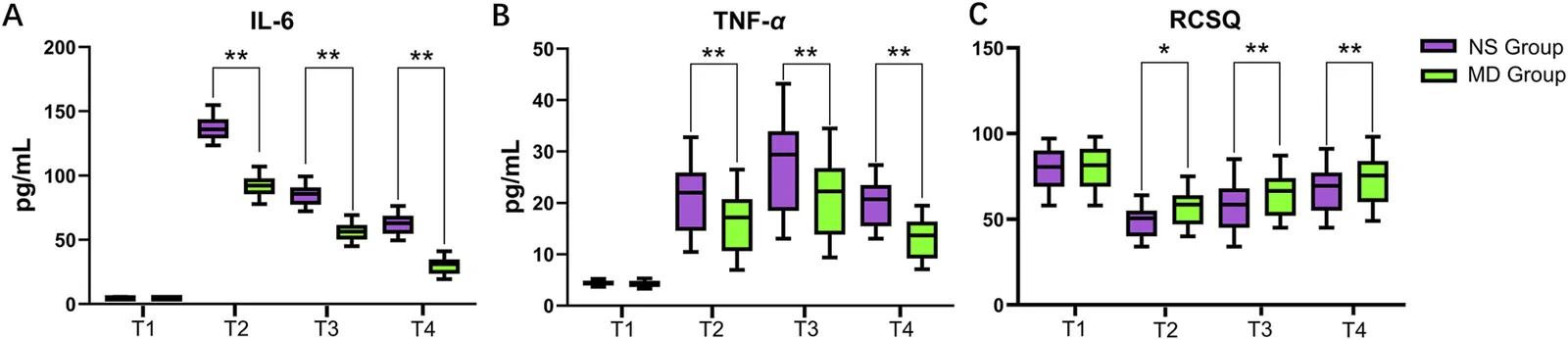
Between-group comparisons of serum IL-6, TNF-α, and RCSQ scores at each time point. From left to right, each represents a different indicator: **(A)** for IL-6, **(B)** for TNF-α, and **(C)** for RCSQ. Data are expressed as the mean ± SD. Independent-samples t-test was used for pairwise comparisons at each time point. *P < 0.05, **P < 0.01, ***P < 0.001 vs. the NS group. IL-6, interleukin-6; TNF-α, tumor necrosis factor-α; RCSQ, Richards-Campbell sleep questionnaire; T1, preoperative; T2, 24 h postoperatively; T3, 48 h postoperatively; T4, 72 h postoperatively.

#### 5.4.2 Adverse events

The incidence of PONV within 72 h was significantly lower in the MD group than in the NS group (9.30% vs. 27.91%; *P* = 0.002). The rate of rescue analgesic use within 72 h postoperatively was also significantly lower in the MD group (20.93% vs. 39.53%; *P* = 0.007). No significant between-group differences were observed in drug-related adverse events, including bradycardia, hypotension, excessive sedation, hypoxemia, or rescue antiemetic use (all *P* > 0.05; Table 5).

**TABLE 5:** Incidence of perioperative complications in the two groups.

Index	NS group (n = 93)	MD group (n = 93)	Value *χ* ^2^	*P*
PONV (%)	27.91	9.30	9.67	0.002*
Rescue analgesia (%)	39.53	20.93	7.41	0.007*
Rescue antiemetics (%)	11.63	9.30	0.29	0.591
Bradycardia (%)	0	3.49	Fisher’s exact test	0.126
Hypotension (%)	0	0	NA	NA
Excessive sedation (%)	0	0	NA	NA
Hypoxemia (%)	0	0	NA	NA

Data are presented as the mean (SD) or n (%); *P < 0.05. SD, standard deviation; PONV, postoperative nausea and vomiting; NA: not applicable.

## 6 Discussion

To the best of our knowledge, this is the first randomized controlled trial exploring the efficacy of multiple low-dose 75 μg intranasal dexmedetomidine administration for the prevention of PPCs in elderly patients undergoing radical thoracoscopic lung-cancer resection. We drew two key conclusions from this study. First, this intervention can markedly lower the incidence of PPCs. Second, it does not raise the risk of related adverse events, confirming that the regimen is safe and effective for PPCs in elderly patients.

A favorable dose–effect relationship has been observed in healthy Chinese adults after a single administration of intranasal dexmedetomidine administration at 25 μg –175 μg (Li et al., 2024). A fixed dose of 100 μg can achieve ideal sedative effects in adults, and the bioavailability estimated by the final model was approximately 65%, with plasma concentrations becoming negligible after 24 h (Li et al., 2024). Based on the above literature combined with our preliminary data, and considering the physiological characteristics of elderly patients, a dose of 75 μg intranasal dexmedetomidine administration administered multiple times was adopted in this study.

This randomized controlled trial demonstrates that multiple low-dose intranasal dexmedetomidine administration reduces the incidence of PPCs in elderly patients undergoing radical thoracoscopic lung-cancer resection. The protective effects may be related to the anti-inflammatory, sedative, and sympatholytic properties of intranasal dexmedetomidine administration, which provide stable and sustained drug exposure throughout the high-risk postoperative period. The non-invasive nasal route improves patient comfort and compliance, especially in elderly populations.

Our study confirmed that the incidence of PPCs in elderly patients undergoing radical thoracoscopic lung-cancer resection was significantly lower than that reported in previous studies (Fernandez-Bustamante et al., 2017a). The possible reasons are as follows: 1) only the incidence of PPCs within 7 days after surgery was considered in this study, with a relatively short observation period (Fernandez-Bustamante et al., 2017a). 2) This study did not enroll elderly patients with high-risk factors for PPCs, such as poor preoperative pulmonary function, severe chronic lung disease, and ASA > III, leading to a lower incidence of PPCs in this study than in previous reports.

In our study, no significant intergroup difference was found in the severity grade of PPCs, and there was insufficient evidence to prove that the intervention could alleviate complication severity after multiple comparison correction. No correction was conducted for the classification of PPCs. Our study found that multiple low-dose intranasal dexmedetomidine administration significantly and continuously inhibited the release of perioperative inflammatory mediators (Dong et al., 2020), improved the sleep quality in elderly hospitalized patients (Wang et al., 2023), and reduced the incidence of PONV (Liu et al., 2025). Accumulating clinical evidence demonstrates various extra-pulmonary perioperative advantages of dexmedetomidine, although delirium, gastrointestinal dysfunction, and arrhythmias were not predefined endpoints. Its favorable effects cover mitigating intraoperative tachycardia and postoperative atrial fibrillation in thoracic surgery patients (Peng et al., 2021; Ma et al., 2025), alleviating stress-induced gastrointestinal dysmotility and postoperative constipation (Huang et al., 2021; Lai et al., 2024; Liu et al., 2024), and preventing postoperative delirium (Zeng et al., 2019; Yu et al., 2022; Na et al., 2023).

This study has several limitations. First, we excluded patients with multiple risk factors for PPCs during participant selection, such as patients who were overweight or obese; therefore, these findings cannot be generalized to elderly patients with multiple relevant risk factors. Second, numerous studies confirm that intravenous or intranasal dexmedetomidine administration confers multifaceted perioperative benefits, such as bronchodilation and lower risks of postoperative delirium, tachycardia, and arrhythmias, including atrial fibrillation. However, these indicators were not assessed in the present study. The clinical value of the intranasal administration regimen adopted in this trial with respect to the above outcomes remains to be verified in further investigations. Third, this was a single-center study. Further large-scale, multi-center trials are warranted to validate efficacy in high-risk patients and define optimal dosing regimens.

## 7 Conclusion

In elderly patients undergoing radical thoracoscopic lung cancer resection, multiple low-dose intranasal dexmedetomidine administration significantly reduces the incidence of PPCs within 7 days after surgery with a favorable safety profile. Further studies are required to verify its efficacy in the elderly population with a wider range of conditions and more underlying diseases.

## Data Availability

All data generated in this study belong to Henan Provincial People’s Hospital. Data sharing is available upon formal request submitted to the research institution and the corresponding author.
