# Supplementary material for: Effects of multiple low-dose intranasal dexmedetomidine administration on postoperative pulmonary complications in elderly patients undergoing thoracoscopic radical resection of lung cancer: a single-center randomized controlled trial
Source: Front Pharmacol. 2026 Oct 2;17:1914046. doi: 10.3389/fphar.2026.1914046 (PMC13672550; doi:10.3389/fphar.2026.1914046)
Supplement: Supplementary file 1 [file Table1.docx]

Supplementary Material

# Supplementary Tables

**Table S1** Standardized PPCs according to the European Perioperative Clinical Outcome Definitions.

| Pneumonia | Patient has received antibiotics for a suspected respiratory infection and met one or more of the following criteria: new or changed sputum, new or changed lung opacities, fever, white blood cell count > 12╳10^9^/L |
| --- | --- |
| Respiratory failure | Postoperative arterial oxygen partial pressure < 8 kPa (60 mmHg) on room air, an arterial oxygen partial pressure to oxygen fraction ratio < 40 kPa (300 mmHg) or arterial oxyhemoglobin saturation measured with pulse oximetry < 90% and requiring oxygen therapy |
| Atelectasis | Lung opacification with a shift of the mediastinum, hilum or hemidiaphragm toward the affected area, and compensatory over-inflation in the adjacent non-atelectatic lung |
| Pulmonary embolism | Diagnosed by CT angiography without severity grading |
| Bronchopleural fistula | Diagnosed by flexible bronchoscopy; persistent requiring re-operation |
| Pleural empyema | Fever, white blood cell count > 12╳10^9^/L and CT scan |

CT, computed tomography; PPCs, postoperative pulmonary complication.

**Table S2** Dynamic changes of IL-6, TNF-*α* and RCSQ in two groups at different time points.

| Index | Time | NS Group (n=93) | MD Group (n=93) | *P* |
| --- | --- | --- | --- | --- |
| IL-6 (pg/mL) | T1 | 4.76±1.02 | 4.80±0.94 | >0.05 |
|  | T2 | 136.63±15.31 | 92.75±11.61 | <0.05 ^abc^ |
|  | T3 | 87.72±12.73 | 59.54±10.44 | <0.05 ^abc^ |
|  | T4 | 64.54±11.54 | 32.12±9.13 | <0.05 ^abc^ |
| TNF-*α* (pg/mL) | T1 | 4.62±0.72 | 4.56±0.86 | >0.05 |
|  | T2 | 22.21±8.53 | 17.53±8.35 | <0.05 ^abc^ |
|  | T3 | 28.80±10.52 | 22.32±9.26 | <0.05 ^abc^ |
|  | T4 | 20.52±5.31 | 13.74±4.74 | <0.05 ^abc^ |
| RCSQ | T1 | 86.12±16.61 | 85.64±16.43 | >0.05 |
|  | T2 | 50.12±9.24 | 58.23±10.45 | <0.05 ^abc^ |
|  | T3 | 59.21±14.95 | 66.47±13.52 | <0.05 ^abc^ |
|  | T4 | 68.68±14.44 | 75.16±15.28 | <0.05 ^abc^ |

**Notes:** Data are presented as mean ± standard deviation. GEE was used for statistical analysis. Main effect: group ^a^ *P*<0.05, time ^b^ *P*<0.05, group×time interaction ^c^ *P*<0.05.

**Abbreviations:** GEE, Generalized estimating equations; IL-6, Interleukin-6; TNF-*α*, Tumor Necrosis Factor-*α*; RCSQ, Richards-Campbell Sleep Questionnaire; T1: preoperative; T2: 24 h after surgery; T3: 48 h after surgery; T4:72 h after surgery.
